# Variability Studies of Two *Prunus*-Infecting Fabaviruses with the Aid of High-Throughput Sequencing

**DOI:** 10.3390/v10040204

**Published:** 2018-04-18

**Authors:** Igor Koloniuk, Tatiana Sarkisova, Karel Petrzik, Ondřej Lenz, Jaroslava Přibylová, Jana Fránová, Josef Špak, Leonidas Lotos, Christina Beta, Asimina Katsiani, Thierry Candresse, Varvara I. Maliogka

**Affiliations:** 1 Department of Plant Virology, Institute of Plant Molecular Biology, Biology Centre of the Academy of Sciences of the Czech Republic, v.v.i., 370 05 České Budějovice, Czech Republic; sarkisova@umbr.cas.cz (T.S.); petrzik@umbr.cas.cz (K.P.); lenz@umbr.cas.cz (O.L.); pribyl@umbr.cas.cz (J.P.); jana@umbr.cas.cz (J.F.); spak@umbr.cas.cz (J.Š.); 2 Laboratory of Plant Pathology, School of Agriculture, Faculty of Agriculture, Forestry and Natural Environment, Aristotle University of Thessaloniki, 54124 Thessaloniki, Greece; llotos@agro.auth.gr (L.L.); chrismpeta@agro.auth.gr (C.B.); meniakats@yahoo.gr (A.K.); vmaliogk@agro.auth.gr (V.I.M.); 3 UMR 1332 Biologie du Fruit et Pathologie, INRA, University of Bordeaux, CS20032, F-33882 Villenave d’Ornon CEDEX, France; thierry.candresse@inra.fr

**Keywords:** plant virus, fabavirus, novel species, prunus, high-throughput sequencing, intrahost variability, phylogenetics

## Abstract

During their lifetime, perennial woody plants are expected to face multiple infection events. Furthermore, multiple genotypes of individual virus species may co-infect the same host. This may eventually lead to a situation where plants harbor complex communities of viral species/strains. Using high-throughput sequencing, we describe co-infection of sweet and sour cherry trees with diverse genomic variants of two closely related viruses, namely prunus virus F (PrVF) and cherry virus F (CVF). Both viruses are most homologous to members of the *Fabavirus* genus (*Secoviridae* family). The comparison of CVF and PrVF RNA2 genomic sequences suggests that the two viruses may significantly differ in their expression strategy. Indeed, similar to comoviruses, the smaller genomic segment of PrVF, RNA2, may be translated in two collinear proteins while CVF likely expresses only the shorter of these two proteins. Linked with the observation that identity levels between the coat proteins of these two viruses are significantly below the family species demarcation cut-off, these findings support the idea that CVF and PrVF represent two separate *Fabavirus* species.

## 1. Introduction

Fruit trees are known to be hosts to a high number of viruses, mainly as a result of their vegetative mode of propagation and long-term cultivation in orchards. Especially in *Prunus*, several new viral species were described in recent years with the aid of high-throughput sequencing (HTS) [1,2]. In 2011, 23 different viruses were known to infect sweet and sour cherry worldwide [3]. This number is constantly increasing and includes viruses belonging to genera that were previously unknown to infect *Prunus* species [4,5]. It was pointed out that mixed infections of *Prunus* sp., and cherry species in particular, are frequent and thus hamper the determination of pathogen-symptoms correlation [1]. Moreover, it was shown that in several occasions the intra-host population of a *Prunus* infecting virus species might be highly heterogeneous, consisting of distant genetic variants [6] with yet unknown implications on the virus pathogenicity and epidemiology.

Existing HTS methodologies already have satisfactory sequencing capacity for the study of such heterogeneous viral populations. For instance, when artificial mixtures of five human immunodeficiency virus 1 genotypes were subjected to HTS, comparable results were produced with different sequencing platforms (Roche 454pyrosequencing, Solexa by Illumina, Single-molecule real-time sequencing by PacBio). In such a situation, the existence of low complexity regions may represent an obvious obstruction for the assembly step of genetically close genotypes [7].

In the present study, we molecularly characterized two closely related viruses of the genus *Fabavirus*, family *Secoviridae*, which were found by HTS to infect cherry trees as mixtures of genotypes. Fabaviruses are single-stranded positive sense RNA viruses with two genomic segments (RNA1 and RNA2). Each RNA encodes a polyprotein which is proteolytically cleaved into the functional proteins. RNA1 contains the nucleoside triphosphate (NTP)-binding, genome-linked protein (VPg), proteinase and polymerase motifs, whereas RNA2 has the movement (MP) and capsid (CP) proteins motifs [8]. During their replication, large protein complexes, associated with endoplasmic reticulum-derived membranes, are formed in infected cells. Fabaviruses have a wide host range among some monocotyledonous and many dicotyledonous families and they are known to be aphid-transmitted in the non-persistent manner [8]. Nevertheless, the genus is not numerous: there are only seven recognized virus species to date. The first fabavirus characterized in sweet cherries and studied herein, prunus virus F (PrVF), was described by Villamor et al. [4] and has recently been reported in the Czech Republic [9]. The second one is presumably a novel virus species, genetically close to PrVF, which was tentatively named cherry virus F (CVF) and is reported here for the first time. CVF was detected in plant materials from the Czech Republic and Greece. The phylogenetic relationships between these two viruses are analyzed and the factors that are putatively shaping their evolution and their frequent existence as mixtures of different variants are discussed.

## 2. Materials and Methods

The majority of samples from cherry trees were collected during 2014–2016 from four locations. Six samples were taken from the germplasm at the Research and Breeding Institute of Pomology, Holovousy, Czech Republic (50.3683644N, 15.5675814E). Two samples, SwC–H and SwC–H6, were collected in South Bohemia region (49.1512472N, 14.4814483E and 49.049139N, 14.4589956E, respectively), approximately 10 km each from the other and 160 km away from the Holovousy village. One sample was collected in 2009, from a sweet cherry orchard in Aridaia, Pella in Greece (Table 1).

Selected specimens were subjected to (a) enrichment of double-stranded RNA (dsRNA) species as described previously [10], (b) isolation of either total RNA and subsequent messenger RNA (polyA) enrichment (MagJET Plant RNA Purification Kit, MagJET mRNA Enrichment Kit, Thermo Scientific, Vilnius, Lithuania) or (c) isolation of small RNAs using mirPremier microRNA Isolation Kit (Sigma-Aldrich, St. Louis, MO, USA) (Table 1).

Enriched dsRNA was extracted following cellulose-based protocol with slight modifications, which included optional S1 nuclease (Thermo Scientific) and TURBO DNase (Life Technologies, Carlsbad, CA, USA) treatments. Quantification and quality controls were done with Nanodrop spectrophotometer and/or Qubit fluorometr and native agarose electrophoresis, respectively. Library preparations were done either externally with TrueSeq Illumina kit or internally with MuSeek Library preparation kit (Thermo Scientific) and subjected to HiSeq2500 sequencing (service provider SeqMe s.r.o., Dobříš, Czech Republic). Small RNAs library construction and sequencing were performed by Life Sequencing S.L. (Paterna, Spain) on an Ion Torrent platform using the Ion 318^TM^ chip (South San Francisco, CA, USA).

The obtained sequence reads were quality trimmed and de novo assembled using CLC Genomic Workbench 8.5.1 (Qiagen, Sverige, Denmark). Verification of assembly consistency was done by back-mapping of trimmed reads onto reference sequences with following parameters—coverage fraction minimum 0.5, identity 0.95. Continuity of the assemblies was visually verified for covering inconsistencies. Total RNA was extracted from 50 mg of fresh leaf material using a MagJET Plant RNA Kit (Thermo Scientific) and transcribed into complementary DNA (cDNA) with random hexamers and Maxima H Minus First Strand cDNA Synthesis Kit (Thermo Scientific) according to the manufacturer’s recommendations. The obtained cDNA was amplified with SapphireAmp Fast PCR Master Mix (Takara, Kusatsu, Japan) and the virus-specific primers (Supplementary Materials Table S1). The products were purified with Sephadex G-50 (GE Healthcare, Sweden) columns and either cloned with pGEM–T Easy system (Promega, USA) or directly sequenced with the corresponding primer by GATC Biotech (Constance, Germany). The cloned products were screened with virus-specific primers and selected plasmid DNAs were sequenced with the plasmid or the virus-specific primers (Supplementary Materials Table S1).

General sequence analyses were done in Geneious 9.1.8 (Biomatters Limited, Auckland, New Zealand). Pairwise comparisons were calculated from CLUSTALW multiple alignments using the matrix percentage tool from the Geneious’s alignment tool. The same approach was applied for comparison of codon positions, which were at first separately extracted using Molecular Evolutionary Genetics Analysis (MEGA) 6.06 software [11] and then spliced. The phylogenetic analysis was performed using MEGA 6.06 software [11]. Sequences were aligned with MUSCLE (MUltiple Sequence Comparison by Log-Expectation) [12] and the phylogenetic tree was reconstructed with the maximum likelihood method using the best substitution model following the Akaike information criterion. All positions with less than 95% site coverage were eliminated. The best substitution model was selected for each dataset separately. Reliability for internal branching was assessed using 500 bootstrap iterations. Synonymous and non-synonymous substitution rates were calculated based on MAFFT (Multiple Alignment using Fast Fourier Transform) codon-aligned datasets using SNAP (Synonymous Non-synonymous Analysis Program) v2.1.1 program [13].

## 3. Results

### 3.1. Sour and Sweet Cherries Host Diverse viruses Belonging to Different Families

High-throughput sequencing analysis of nine sweet or sour cherry samples originating from the Czech Republic and Greece revealed the presence of several viruses (Table 2), three of which belong to novel species: one belongs to the family *Luteoviridae*, and other two to the family *Secoviridae*.

In the present study, we investigated the later two, both verified by Sanger sequencing with appropriate primers (Supplementary Materials Table S1). Detailed analysis revealed that the first of the two viruses was highly identical to PrVF (up to 97% and 98% of overall nucleotide identities of RNA1 and RNA2, respectively), which was reported during the course of our study [4]. For this virus, we obtained the complete sequences of three sets of genomic RNAs (Table 3) from the Sweet cherry sample 43 (SwC–43), which was infected by variants representing all the molecular groups of PrVF, as discussed below. The second novel virus was present in three samples (SC–46, SwC–H, and G15–3). It is highly similar to PrVF, (up to 70% and 59% overall nucleotide identities of RNA1 and RNA2, respectively), however, below the secovirids’ species demarcation level (details in Section 3.5). The virus was tentatively named CVF. PrVF showed higher intra-host variability than CVF, for which mixed infection by two different variants (of RNA1) was only detected in the SwC–H sample (Table 3).

The inter-sample molecular similarities of PrVF genotypes allowed their clustering (names of clusters for RNA1 and RNA2 were chosen arbitrary) into several groups of molecular identity (three for each RNA: A, B, C in further text) supported as well by phylogenetic analyses. It is not clear at this stage if preferential associations exist between the genomic RNAs 1 and 2 of PrVF belonging to different clusters. In the majority of PrVF-positive samples RNA1 B and RNA2 B were present. All samples contained more than one variant of RNA1, whereas three of them contained a single variant of RNA2. Samples SwC–H and SwC–H6, hosted two variants of RNA1 from the same molecular group (Table 3).

### 3.2. Comparison of CVF and PrVF Genome Organization

**Figure 1:**
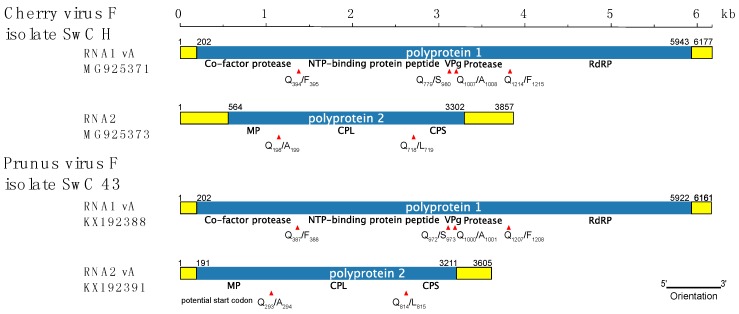
Genome organization of CVF and PrVF. The predicted cleavage sites and their positions are shown by red triangles with annotated amino acids. Untranslated regions are shown as yellow filled boxes. The nucleotide positions of genomic elements are marked at the extremities. The segments are drawn to scale. MP: movement protein; CPL: large capsid protein; CPS: small capsid protein; NTP: nucleoside triphosphate; VPg: genome-linked protein.

**Figure 2:**
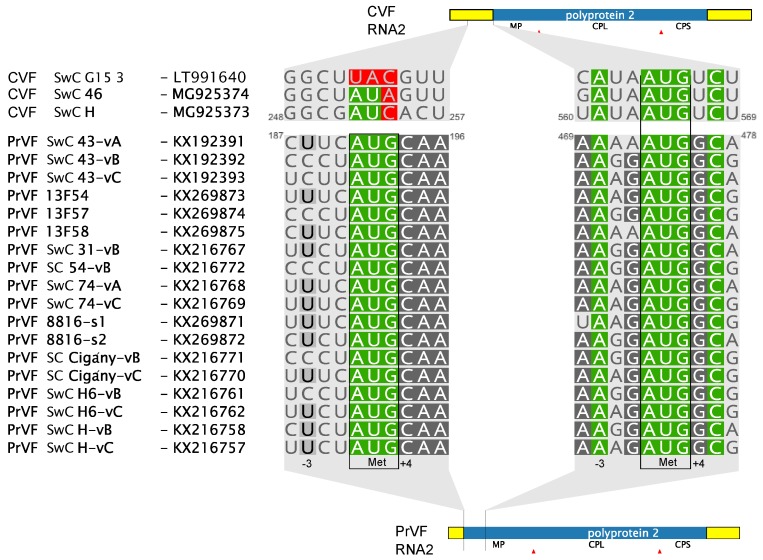
Prediction of start codons for RNA2—encoded polyproteins. The left alignment shows PrVF initiation codon predicted by Villamor et al. [4], with the corresponding mutated bases in the CVF sequence highlighted in red. The right alignment displays proposed translation initiation codon of PrVF based on the CVF sequence and on the more efficient context for translation initiation.

As for other fabaviruses, the genome of CVF–H is divided between two genomic segments (Figure 1). The organization is similar to members of the *Fabavirus* genus and, particularly, to that of PrVF. The sequences of two variants of the RNA1 segment, 6169 nucleotides (nt) and 6188 nt long, respectively, and of one 3857 nt long RNA2 segment were obtained. Further, each 3′ terminus had polyA tail of undefined length. The lengths of both RNAs are comparable with those of PrVF and other fabaviruses. RNA1 of CVF is among the longest species, with an exception of RNA1 of peach leaf pitting-associated virus that is 6357 nt long [2]. Both CVF and PrVF genomic RNAs contain a 10 nt long conserved region 5′-AACCGCUUUC-3′ at the 5′ untranslated region (UTR), a sequence similar to the one reported for other fabaviruses (5′-AACAGCUUUC-3′, the single mutation separating these conserved regions is underlined) [4,14]. A notable difference between CVF and PrVF is that the predicted MP of CVF is 91 amino acids (aa) shorter than that of PrVF [4]. This results in a 373 nt longer 5′ UTR for the CVF RNA2 segment. The analysis of a multiple alignment of available PrVF RNA2 sequences revealed that in a close vicinity to that of CVF’s AUG codon there is another potential PrVF start codon downstream of the previously described one (Figure 1). This second start codon of PrVF (Figure 2) is in a more favorable context for translation initiation, according to the Kozak’s rule [15], when compared to the start codon of CVF RNA2, which has a uracil residue at the +4 position (Figure 2).

No AUG codon was found upstream of the CVF MP, however, there are AUA or AUC codons, in frame with the following polyprotein and approximately in the same alignment position as the first start codon of PrVF (Figure 2). This could be a mutated start codon of a former longer polyprotein of CVF. Although the region between the supposedly mutated and the predicted start codons of all three CVF isolates is less conserved than the MP (62.9% and 91.6% of aa identity, respectively), this region does not contain any in-frame stop codons suggesting an evolutionary recent mutation of the start codon and/or some kind of selection conserving the sequence within this area.

The putative cleavage sites of CVF polyproteins were compared with those predicted for PrVF and were found to be identical, with the exception of the cleavage site between the large and small CPs (Figure 1).

### 3.3. Phylogenetic Inference

**Figure 3:**
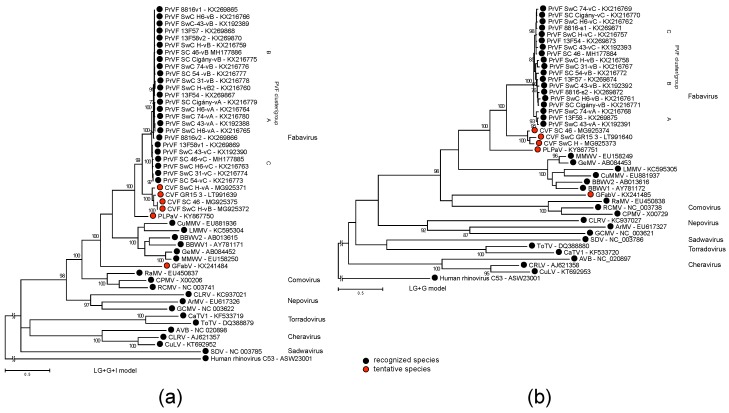
Maximum Likelihood phylogenetic tree of (**a**) CG–GDD region (ProPol), and (**b**) coat proteins domains of CVF, PrVF, and selected members of the family *Secoviridae*: Satsuma dwarf virus (SDV), arracacha virus B (AVB), cherry rasp leaf virus (CRLV), currant latent virus (CuLV), cherry leaf roll virus (CLRV), arabis mosaic virus (ArMV), grapevine chrome mosaic virus (GCMV), tomato torrado virus (ToTV), carrot torradovirus 1 (CaTV1), radish mosaic virus (RaMV), red clover mottle virus (RCVMV), cowpea mosaic virus (CPMV), grapevine fabavirus (GFabV), lamium mild mosaic virus (LMMV), cucurbit mild mosaic virus (CuMMV), broad bean wilt virus 1 (BBWV1), broad bean wilt virus 2 (BBWV2), gentian mosaic virus (GeMV), mikania micrantha wilt virus (MMWV, an isolate of GeMV), peach leaf pitting-associated virus (PLPaV), prunus virus F (PrVF), cherry virus F (CVF). The substitution model is shown above each tree. The sequence of human rhinovirus C53, an enterovirus, was used as an outgroup. Support values for partitions supported by less than 70% of bootstrap replicates are not shown.

The sequence of the region between the CG motif of protease to the GDD motif of RNA polymerase and the sequence of the CPs were used for phylogenetic analyses of CVF, PrVF and selected representative members of the *Secoviridae* family (Figure 3). Both analyses placed CVF and PrVF as separate entities within the genus *Fabavirus* (Figure 3).

### 3.4. Analysis of Recombinants

Genetic algorithm recombination detection (GARD) and single breakpoint (SBP) recombination analyses found several potential recombination breakpoints in the RNA1 of PrVF but none of them was characterized by significant topological incongruence (*p* = 0.05). Further analyses with RDP4 (Recombination detection program) [16] revealed two recombination events in PrVF sequences supported by the RDP, GENECONV, Bootscan, Maxchi, Chimaera, SiSscan, and 3Seq methods implemented in the software. The first predicted recombination took place in the RNA1 protease-encoding region while the other one lies in the 3′ UTR of RNA2 (Supplementary Materials Table S2).

### 3.5. Genetic Diversity among Different Isolates of PrVF and CVF and Other Fabaviruses

**Figure 4:**
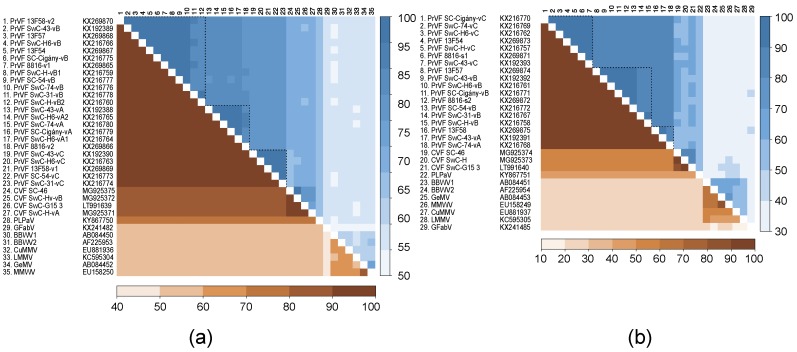
Pairwise comparison of CG–GDD (**a**) and capsid proteins (**b**) identities-nucleotide and aminoacid sequence identities are shown above and below the diagonal, respectively.

Figure 4 shows pairwise identity comparisons for the CG–GDD (Pro–Pol) region and the CPs between different isolates of CVF and PrVF and other fabaviruses. There is evident segregation of isolates into three Pro–Pol clusters and three CP clusters for PrVF. According to the currently applied criteria for species demarcation in the *Secoviridae* family, isolates of distinct species share less than 80% and 75% of aa identities in the Pro–Pol and CP regions, respectively [8,17,18]. The divergence of the CPs between CVF and PrVF is higher than for the Pro–Pol region, reaching identity levels of only 50–60%, values that are well below the 75% species cut-off. At the same time, several isolates of PrVF were up to 84% identical with CVF isolates in the Pro–Pol region, while all comparisons provided values in excess of the 80% species cut-off. There is apparent separation of PrVF, CVF, PLPaV from the other fabaviruses in both analyzed regions: The aa identities were not higher than 60% for Pro–Pol and 40% for CP regions.

**Figure 5:**
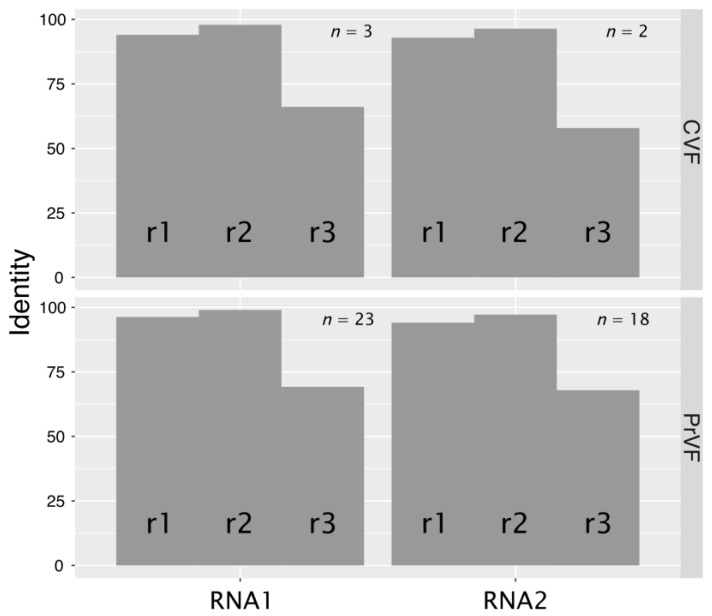
Comparison of variability of different positions in CVF and PrVF codons. Nucleotide sequences encoding polyproteins were codon-aligned with MAFFT (Multiple Alignment using Fast Fourier Transform) [20]. Every first, second and third position was extracted into individual dataset and respective identities were calculated. Numbers of analyzed sequences (*n*) are shown above each codon.

Levels of nt conservation between sequence variants of CVF or PrVF identified within individual plant samples were strikingly low, on average less than 80%, whereas the translated aa sequences were more conserved (Figure 4). Further analysis showed that the majority of changes were synonymous. The ratios between nonsynonymous and synonymous changes (dN/dS) were calculated to be 0.012 and 0.041 for RNA1 (*n* = 3) and RNA2 (*n* = 2) of CVF, respectively. The obtained values for PrVF were similar: 0.012 (RNA1, *n* = 23) and 0.047 (RNA2, *n* = 18). Substitution rates at the three codon positions (r1, r2, and r3) are in the order r3 > r1 > r2 (Figure 5). Analysis of substitutions between different variants of genomic RNA of CVF and PrVF revealed a strong bias toward the conservation at the r2 position, that likely reflects the invariably nonsynonymous nature of the second position substitution being under evolutionary constraint [19].

## 4. Discussion

Cherry virus F, together with prunus virus F and the recently described peach leaf pitting-associated virus [2] form a distinct *Prunus*-infecting phylogenetic cluster within the *Fabavirus* genus. Given their extensive spread (North America, Asia and Europe) [2,4,8] and the significant genetic divergence observed between them it is possible that they have not emerged due to a recent host jump, but rather that they represent a neglected group of viruses, which has been infecting *Prunus* for a long time.

CVF likely represents a new virus species, close to PrVF. These viruses exhibit low identity levels with the other fabaviruses and form a separate phylogenetic cluster (Figure 3). The use of the currently applied species demarcation cut-off values to separate CVF and PrVF is not straightforward, because while CPs comparisons fall clearly outside of the species boundary, which is set as 75% or less of aa identity, comparisons for the Pro-Pol region fall close or slightly within the boundary (80% or less). Similar situations have previously been observed in the *Secoviridae* family, for example in the case of Tomato black ring virus (TBRV) and Beet ringspot virus (BRSV) in the *Nepovirus* genus [18]. In this case, also a low CP identity (62%) and a high Pro-Pol identity (89%) was observed, which, however, given some serological differences, did not prevent the separation of these agents in distinct species [18]. Besides sequence comparison, the species demarcation criteria list other properties as absence of cross-protection and re-assortment between RNA segments, distinct host range and vector, and serological differences [18].

In the case of CVF, a distinct difference with PrVF seems to be the presence of an exceptionally long RNA2 5′ UTR and the inability to encode the long version of the polyprotein that can be produced by PrVF. The existence of two in-frame initiation codons is a feature known in related comoviruses. For cowpea mosaic virus (genus *Comovirus*, family *Secoviridae*) it has been shown that the upstream AUG is in a suboptimal context and that it may be bypassed during ribosomes scanning so that translation is also initiated from a downstream start codon in an optimal context [21]. The RNA2 of Bean pod mottle virus (BPMV, genus *Comovirus*, family *Secoviridae*) is translated in a similar fashion producing two proteins differing in their C’ part: P58 and MP [22]. Moreover, a pivotal role of the CPMV downstream starting codon in viral cell-to-cell movement has been demonstrated [21], while mutation of the upward AUG in RNA2 of BPMV led to abolishment of the virus replication [22]. Thus, we may speculate about the existence of a similar situation in the case of PrVF RNA2 translation, yielding two distinct, co-linear polyproteins, while in contrast the RNA2 of CVF with its single predicted start codon would only encode the shorter version of the polyprotein (Figure 1 and Figure 2).

In this study, we highlight the ability of CVF and PrVF to co-infect cherry hosts as a complex mixture of variants (Table 3). A similar situation was also reported by Villamor et al. during the initial description of PrVF [4]. While mixed infections are frequent in fruit tree species, the fact that simple infection of a cherry tree by a single PrVF variant appears to be very rare is remarkable. Viral sequence variants were named here as *variants* or *genotypes* interchangeably as these terms are the closest to the description of their nature. Viral *strains* may not represent an appropriate definition as they are based on certain, usually biological or serological, differences. It appears that for the limited number of analyzed samples the prevalence of PrVF infections was higher than that of CVF (Table 1), at least in the Czech Republic. Further, the number of identified PrVF genotypes is also higher than for CVF, which were detected only in the Czech samples (Table 2 and Table 3).

The generation of numerous viral mutants during replication is an important driving force of virus evolution, although the majority of them are suggested to be non-infectious or to have reduced fitness [23]. Several studies considered viral variants to form a conglomeration of virus subpopulations that are segregated and spatially structured within a plant [24,25]. This could reduce both intercellular competition for host resources between different viral variants and natural selection efficiency as more virus variants with lower fitness may persist within as separated subpopulations [26]. The viral variants form a spectrum surrounding a *master* genotype sequence, the one with the highest replication rate, and could be segregated into major and minor frequency clusters [27]. Mixed infections of a single tree with multiple variants, a specific trait which for some reason is highly pronounced in PrVF, might be linked to unknown epidemiological characteristics. Clustering of variants originating from different countries (Figure 3 and Figure 4), could indicate the multiple influxes of viruses either from neighbouring cherry producing countries or by long distance movement of infected propagative material. Fabaviruses are transmitted non-persistently by aphids, which could contribute to a rapid spread of a virus, although natural vectors of CVF and PrVF, if there exists any, are yet to be identified.

From an ecological point of view, it is not so clear how such diverse populations of viruses could sustain a co-infection of their hosts. Sympatric viral genotypes represent operational taxonomic units with overlapping geographic distribution. It was suggested that such coexistence required effective separation barrier, either biotic or reproductive [28] otherwise leading to superinfection cases. While there are no broad studies of superinfection incidences for different plant viruses, PrVF was found to infect cherry trees almost exclusively (with the exception of samples 13F54 and 13F57 [4]) as a mixture of genotypes in different geographic locations. Existing analyses of superinfection cases suggested that sequence homology plays important role during colonization of the host with multiple viral genotypes [29,30]. That is in accordance with the observed intra-host variability of both PrVF and CVF genomes. At the same time, degeneracy of the genetic code brings a multitude of nucleotide combinations for any protein sequence, forming sequence space, a geometric representation of all possible mutants [31]. Available PrVF genotypes may be segregated into several phylogenetic lineages (Figure 3 and Figure 4), thus pointing to efficient selective constrains that allowed occupation of a limited part of the sequence space.

Beside high mutation rates, two other mechanisms are responsible for genetic variation in RNA viruses: recombination and reassortment [32]. Having analyzed available PrVF and CVF sequences, only two recombination signatures were found in one German and one American isolates. However, such regions need to be reanalyzed to exclude a possibility of incorrect assembly step, as the 13F58-v1 isolate of PrVF was predicted to involve the 13F58-v2 isolate from the same sample as its minor parent (Supplementary Materials Table S2).

The observation that PrVF and possibly CVF genotypes are present in arbitrary mixtures of both genomic segments among the sampled trees is particularly striking in this respect, as these genotypes are phylogenetically distinct and do not show significant evidences of multiple recombination events. It is unclear if generally the viral replication machinery originating from one genotype distinguishes other RNA genotype(s) or processes them with equal efficiency as the original RNA template.

With the broader application of HTS in plant pathology, a deeper insight into intra-host variability of phytoviruses will be obtained in the near future. Understanding mechanisms of establishment and successful maintenance of intra-host heterogeneous genotypes may lead to discovery of novel virus–virus interactions and subsequent impact on disease phenotype. Given the high number of viral agents detected in the analyzed samples in this study it is not possible to evaluate the contribution of distinct species and/or their combinations to the observed symptoms, especially when there were also cases of no apparent manifestations (Table 1). Single infections of cherry trees with the herein described fabaviruses would help to clarify whether these viruses are able to cause symptoms. The direction of further research should focus on interactions of RNAs from distinct molecular groups within the same tissues and cells. It is still not clear which mechanisms secure preservation of individual RNAs during such infections.

## Figures and Tables

**Table 1:** Description of samples and applied enrichment for library preparation.

Sample	Description	Origin	Applied Enrichment	Symptoms	HTS, Length of Reads and Output (Millions)
SwC–31	Sweet cherry cv Kisinevskaja,	Chișinău, Moldova	dsRNA	No obvious symptoms	100b, 24
SwC–43	Sweet cherry cv Lambert	Canada	dsRNA	Premature leaf fall, dieback, bunches of small leaves and blossoms growing at the old branches	100b, 25
SC–54	Sour cherry cv Dneprovka	Chișinău, Moldova	dsRNA	Dwarfism	100b, 23
SC–46	Sour cherry cv Rannaja	Chișinău, Moldova	dsRNA	No obvious symptoms	100b, 24
SwC–74	Sweet cherry cv Rube	Magdeburg, Germany	dsRNA	Dwarfism	100b, 25
SC–Cigány	Sour cherry, unknown cultivar	Hungary	dsRNA	Severe mosaic	100b, 25
SwC–H	Sweet cherry, unknown cultivar	Czech Republic	polyA	No obvious symptoms, old age	100b, 20
SwC–H6	Sweet cherry, unknown cultivar	Czech Republic	polyA	No obvious symptoms, old age	100b, 39
SwC–G15 3	Sweet cherry cv tragana-edessis	Greece	sRNAs	No obvious symptoms, old age	21b-24b, 2

HTS: high-throughput sequencing; dsRNA: double-stranded RNA; sRNA: small RNAs; polyA: messenger RNA.

**Table 2:** Mixed viral infections were documented for sweet (SwC) and sour (SC) cherry samples.

	Samples								
	SwC–31	SwC–43	SC–46	SC–54	SwC–74	SC–Cigány	SwC–H	SwC–H6	SwC–G15 3
*Betaflexiviridae*									
Apple chlorotic leaf spot virus									
Cherry green ring mottle virus									
Cherry necrotic rusty mottle virus									
Cherry virus A									
*Bromoviridae*									
Prune dwarf virus									
Prunus necrotic ringspot virus									
*Closteroviridae*									
Little cherry virus 1									
Little cherry virus 2									
*Luteoviridae*									
Cherry–associated luteovirus									
Cherry–associated luteovirus 2 (Thierry Candresse et al. Novel luteovirus infecting sour cherry, submission data preparation)									
*Secoviridae*									
Cherry leaf roll virus									
Prunus virus F									
Cherry virus F									
*Tymovirales*									
*Grapevine Syrah virus 1*-like virus									

**Table 3:** Number and phylogenetic group (in parentheses, with corresponding accession number) of the variants of each genomic RNA of cherry virus F (CVF) and prunus virus F (PrVF) detected in infected cherry samples (na—not resolved). For genetic identities between genomic variants see Supplementary Materials Figure S1 and Figure 4.

Sample	Virus	Variants	
		RNA1	RNA2
SwC–31	PrVF	2 (B–KX216778, C–KX216774)	1 (B–KX216767)
SwC–43	PrVF	3 (A–KX192388, B–KX192389, C–KX192390)	3 (A–KX192391, B–KX192392, C–KX192393)
SC–54	PrVF	2 (B–KX216777, C–KX216773)	1 (B–KX216772)
SwC–74	PrVF	2 (A–KX216780, B–KX216776)	2 (A–KX216768, C–KX216769)
SC–Cigány	PrVF	2 (A–KX216779, B–KX216775)	2 (B–KX216771, C–KX216770)
SC–46	PrVF	2 (B–MH177886, C–MH177885)	1 (C–MH177884)
	CVF	1 (na–MG925375)	1 (na–MG925374)
SwC–H	PrVF	2 (B–KX216759, B–KX216760)	2 (B–KX216758, C–KX216757)
	CVF	2 (A–MG925371, B–MG925372)	1 (na–MG925373)
SwC–H6	PrVF	2 (A–KX216764, A–KX216765, B–KX216766, C–KX216763)	1 (B–KX216761, C–KX216762)
SwC–G15 3	CVF	1 (na–LT991639)	1 (na–LT991640)

## Supplementary Materials

The following are available online at http://www.mdpi.com/1999-4915/10/4/204/s1, Figure S1: Pairwise comparison of PrVF and CVF isolates, Table S1: List of used primers and expected sizes of PCR amplified bands with the above-listed primers, Table S2: Recombination analysis of CVF and PrVF isolates.
