# Supplementary material for: Variability Studies of Two Prunus-Infecting Fabaviruses with the Aid of High-Throughput Sequencing
Source: Viruses. 2018 Apr 18;10(4):204. doi: 10.3390/v10040204 (PMC5923498; doi:10.3390/v10040204)
Supplement: Supplementary file 1 [file viruses-10-00204-s001.zip › Supplementary Figure 1.pdf]

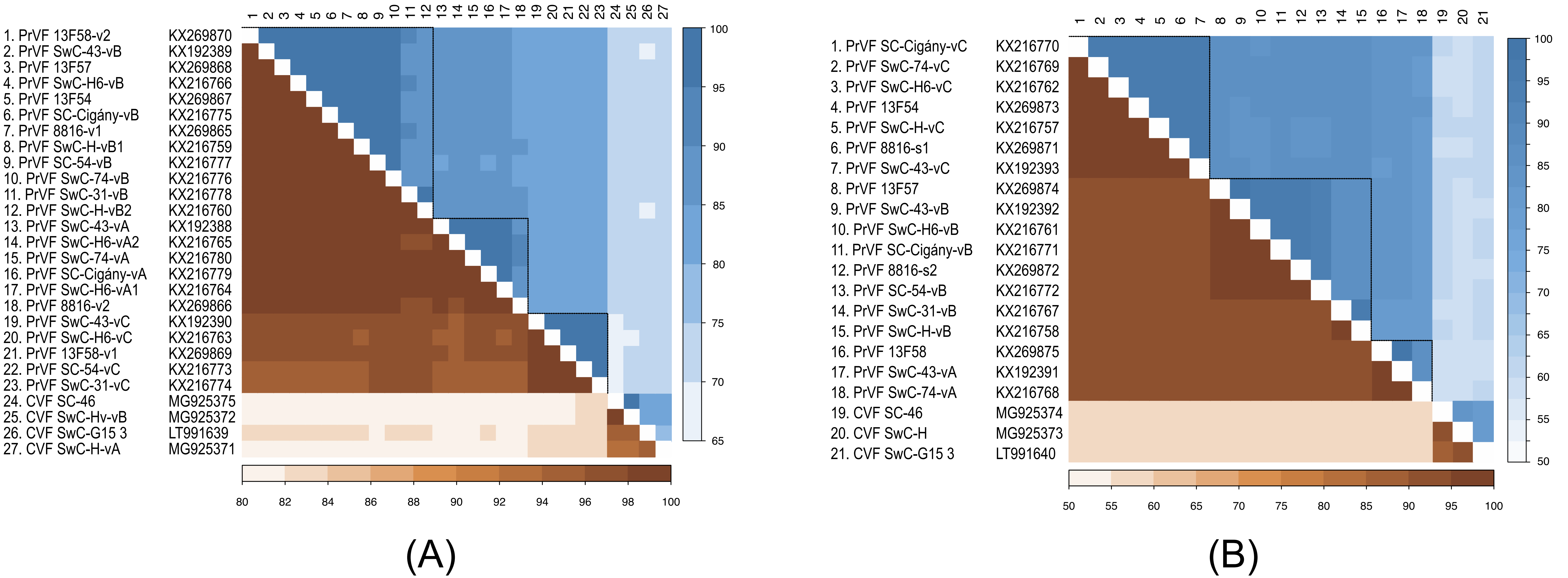

Supplementary Figure 1 Pairwise comparison of CG–GDD (A) and CP (B) identities – nucleotide and aminoacid sequence identities are shown above and below the diagonal, respectively.
