# Supplementary material for: Variability Studies of Two Prunus-Infecting Fabaviruses with the Aid of High-Throughput Sequencing
Source: Viruses. 2018 Apr 18;10(4):204. doi: 10.3390/v10040204 (PMC5923498; doi:10.3390/v10040204)
Supplement: Supplementary file 1 [file viruses-10-00204-s001.zip › Supplementary Table 1.pdf]

Supplementary Table 1 List of used primers. Each binding site is listed

Name	Target	Accession number	Position	Direction	Usage	Sequence, 5′ - 3′ direction
**1007**	CVF SwC–H RNA1B	MG925372	336 – 357	reverse	5' RACE	GATGCCTTTTGTACTTTCTCAT
**1009**	PrVF SwC–43 RNA2C	KX192393	903 – 922	reverse	sequencing	ATTTTGTGCAACCAATCCGT
**1010**	PrVF SwC–43 RNA2C	KX192393	805 – 823	reverse	sequencing	CATGGCCATAACCACTGGA
**1010**	PrVF SwC–43 RNA2B	KX192392	828 – 846	reverse	sequencing	CATGGCCATAACCACTGGA
**1011**	PrVF SwC–43 RNA2B	KX192392	757 – 776	reverse	sequencing	TGATTGCCACCAAGTGCTGT
**1012**	PrVF SwC–43 RNA2B	KX192392	481 – 502	reverse	5' RACE	GRAAYGCCATCYTTTCAAGCTT
**1012**	PrVF SwC–43 RNA2C	KX192393	458 – 479	reverse	5' RACE	GRAAYGCCATCYTTTCAAGCTT
**1012**	PrVF SwC–43 RNA2B	KX192392	481 – 502	reverse	5' RACE	GRAAYGCCATCYTTTCAAGCTT
**1012**	PrVF SwC–43 RNA2C	KX192393	458 – 479	reverse	5' RACE	GRAAYGCCATCYTTTCAAGCTT
**1013**	PrVF SwC–43 RNA2B	KX192392	3182 – 3202	forward	3' RACE	GAAGTGTACCCAAAACAACTA
**1014**	PrVF SwC–43 RNA2A	KX192391	891 – 910	reverse	sequencing	ACCATCCACCAAAATCTCTA
**1015**	PrVF SwC–43 RNA1C	KX192390	642 – 664	reverse	5' RACE	CTGTGCACTTTTCATAGATCACA
**1016**	PrVF SwC–43 RNA1C	KX192390	5300 – 5319	forward	sequencing	AGGAGTGCATGGACCTACGT
**1017**	PrVF SwC–43 RNA1B	KX192389	798 – 818	reverse	5' RACE	TATGCTCCGCCCAGAGCTTGA
**1018**	PrVF SwC–43 RNA1A	KX192388	862 – 881	reverse	5' RACE	AGACCCATAACAACACTATC
**1018**	PrVF SwC–43 RNA1B	KX192389	857 – 876	reverse	5' RACE	AGACCCATAACAACACTATC
**1019**	PrVF SwC–43 RNA1A	KX192388	803 – 825	reverse	5' RACE	TATATGTTCTGCCCAAAGTTTAA
**1020**	PrVF SwC–43 RNA1A	KX192388	613 – 635	reverse	5' RACE	AAAGATGCACAYGARGACATAAG
**1020**	PrVF SwC–43 RNA1C	KX192390	601 – 623	reverse	5' RACE	AAAGATGCACAYGARGACATAAG
**1020**	PrVF SwC–43 RNA1B	KX192389	608 – 630	reverse	5' RACE	AAAGATGCACAYGARGACATAAG
**1029**	PrVF SwC–43 RNA2C	KX192393	268 – 289	reverse	5' RACE	CTCCTCGTATTCAAGCCAAGTT
**1030**	PrVF SwC–43 RNA2B	KX192392	318 – 339	reverse	5' RACE	CACGAACGTGGATGTGTAATCA
**1031**	PrVF SwC–43 RNA2A	KX192391	301 – 321	reverse	5' RACE	GACACAAACGTAAAGGTTCGA
**1034**	PrVF SwC–43 RNA1B	KX192389	990 – 1012	reverse	5' RACE	AGCTTTRTAWCCACATATAGCTC
**1034**	PrVF SwC–43 RNA1A	KX192388	995 – 1017	reverse	5' RACE	AGCTTTRTAWCCACATATAGCTC
**1037**	PrVF SwC–43 RNA1B	KX192389	5 – 27	forward	nearly complete segment amplification	TAAGAGATTAAACAACCGCTTTC
**1037**	PrVF SwC–43 RNA1C	KX192390	5 – 27	forward	nearly complete segment amplification	TAAGAGATTAAACAACCGCTTTC
**1037**	PrVF SwC–43 RNA2C	KX192393	5 – 27	forward	nearly complete segment amplification	TAAGAGATTAAACAACCGCTTTC
**1037**	PrVF SwC–43 RNA2A	KX192391	5 – 27	forward	nearly complete segment amplification	TAAGAGATTAAACAACCGCTTTC
**1037**	PrVF SwC–43 RNA2B	KX192392	5 – 27	forward	nearly complete segment amplification	TAAGAGATTAAACAACCGCTTTC
**1037**	PrVF SwC–43 RNA1A	KX192388	5 – 27	forward	nearly complete segment amplification	TAAGAGATTAAACAACCGCTTTC
**1038**	PrVF SwC–43 RNA2C	KX192393	3725 – 3744	reverse	nearly complete segment amplification	GCTTTCACCAATTCTCAACA
**1038**	PrVF SwC–43 RNA1A	KX192388	6132 – 6151	reverse	nearly complete segment amplification	GCTTTCACCAATTCTCAACA
**1038**	PrVF SwC–43 RNA2A	KX192391	3571 – 3590	reverse	nearly complete segment amplification	GCTTTCACCAATTCTCAACA
**1038**	PrVF SwC–43 RNA1B	KX192389	6134 – 6153	reverse	nearly complete segment amplification	GCTTTCACCAATTCTCAACA
**1038**	CVF SwC–H RNA1B	MG925372	6157 – 6176	reverse	nearly complete segment amplification	GCTTTCACCAATTCTCAACA
**1038**	CVF SwC–H RNA2	MG925373	3828 – 3847	reverse	nearly complete segment amplification	GCTTTCACCAATTCTCAACA
**1038**	PrVF SwC–43 RNA2B	KX192392	3434 – 3453	reverse	nearly complete segment amplification	GCTTTCACCAATTCTCAACA
**1039**	CVF SwC–H RNA2	MG925373	139 – 159	forward	5' RACE	TGGTTACCAAAGCCCTCATTT
**1083**	PrVF SwC–43 RNA1A	KX192388	103 – 120	forward	nearly complete segment amplification	GTGGTTACTCGCTGCCTT
**1083**	PrVF SwC–43 RNA1B	KX192389	98 – 115	forward	nearly complete segment amplification	GTGGTTACTCGCTGCCTT
**1083**	PrVF SwC–43 RNA1C	KX192390	95 – 112	forward	nearly complete segment amplification	GTGGTTACTCGCTGCCTT
**1150**	PrVF SwC–43 RNA1C	KX192390	3408 – 3430	forward	sequencing	GGATAGTGAACTTGCAGTATATC
**1406**	CVF SwC–H RNA1B	MG925372	369 – 388	reverse	5' RACE	GTGCACCCAAGAAACATCTT
**1407**	CVF SwC–H RNA2	MG925373	282 – 301	reverse	5' RACE	TCCTCAACTGTCTCGTACTC
**1408**	CVF SwC–H RNA2	MG925373	382 – 401	reverse	5' RACE	AACAAAGGAATGAGCAACGT
**1409**	CVF SwC–H RNA2	MG925373	848 – 867	reverse	sequencing	AAGGAACAGGCACATCAATC
**1445**	CVF SwC–H RNA2	MG925373	274 – 295	reverse	5' RACE	ACTGTCTCGTACTCTCCCTTGA
**1446**	CVF SwC–H RNA1A	MG925371	5890 – 5911	forward	3' RACE	CAGCAAACAAAGAATCTGGGAT
**1447**	CVF SwC–H RNA1B	MG925372	222 – 242	reverse	5' RACE	AGAACCATCAGAGTGAATGCT
**1448**	CVF SwC–H RNA1A	MG925371	429 – 449	reverse	5' RACE	TCTGAGACAAGAAGGGTTGGA
**1449**	CVF SwC–H RNA1B	MG925372	5837 – 5856	forward	3' RACE	TTGTGGAGCCTTGGCTGTAG
**1450**	CVF SwC–H RNA1A	MG925371	5984 – 6003	forward	3' RACE	TTCCAGTGTGTTGGCAGACA
**1450**	CVF SwC–H RNA1B	MG925372	6004 – 6023	forward	sequencing	TTCCAGTGTGTTGGCAGACA
**1450**	CVF SwC–H RNA2	MG925373	3354 – 3373	forward	3' RACE	TTCCAGTGTGTTGGCAGACA
**1451**	CVF SwC–H RNA2	MG925373	3281 – 3300	forward	sequencing	TGTCAGCATTGCCACTCTCT
**1452**	CVF SwC–H RNA1B	MG925372	138 – 156	reverse	5' RACE	GAAATGAGGGCTTTGGTAA
**1452**	CVF SwC–H RNA1A	MG925371	163 – 181	reverse	5' RACE	GAAATGAGGGCTTTGGTAA
**1542**	CVF SwC–H RNA2	MG925373	589 – 608	reverse	5' RACE	AGATGTTATAGTGCGAGAAG

**1543**	CVF SwC–H RNA1A	MG925371	303 – 323	reverse	5' RACE	TCATCAAGATCCACAATTCCC
**1544**	CVF SwC–H RNA1A	MG925371	223 – 241	reverse	5' RACE	CACAATCAGCTGGAACACA
**1562**	CVF SwC–H RNA2	MG925373	1614 – 1633	forward	sequencing	GAAGTTGAGATGAGTATGCC
**1632**	CVF SwC–H RNA1A	MG925371	100 – 119	reverse	5' RACE	TCAACCAAAGAGCAGACGAA
**818**	PrVF SwC–43 RNA2B	KX192392	3153 – 3173	reverse	sequencing	CCTCCAGCACTTCCAGAAACA
**819**	PrVF SwC–43 RNA2B	KX192392	2621 – 2644	forward	sequencing	GTGGATACCCTTTCTCTGCATCTC
**820**	PrVF SwC–43 RNA1A	KX192388	4411 – 4431	reverse	sequencing	ACACTCGATTCCCACCAACTC
**821**	PrVF SwC–43 RNA1A	KX192388	4238 – 4262	reverse	sequencing	CCTTCCGAAGTCCCTATCACTAGAG
**822**	PrVF SwC–43 RNA1A	KX192388	3983 – 4004	forward	sequencing	CCTGTGAAAAGATACCCAGCGT
**823**	PrVF SwC–43 RNA1A	KX192388	3651 – 3673	forward	sequencing	GATACCTCGTTTCATGACCTACG
**827**	PrVF SwC–43 RNA2C	KX192393	220 – 238	forward	5' RACE	GGGCTTTGAAACATACGCT
**827**	PrVF SwC–43 RNA2C	KX192393	220 – 238	forward	5' RACE	GGGCTTTGAAACATACGCT
**829**	PrVF SwC–43 RNA2B	KX192392	1426 – 1446	forward	sequencing	CTTCAATTTGACAATCCGAAC
**829**	PrVF SwC–43 RNA2C	KX192393	1403 – 1423	forward	sequencing	CTTCAATTTGACAATCCGAAC
**830**	PrVF SwC–43 RNA2B	KX192392	1421 – 1442	reverse	sequencing	GGATTGTCAAATTGAAGAACAA
**830**	PrVF SwC–43 RNA2C	KX192393	1398 – 1419	reverse	sequencing	GGATTGTCAAATTGAAGAACAA
**833**	PrVF SwC–43 RNA1B	KX192389	5828 – 5845	forward	sequencing	AGGGAGGATTCGAACGTC
**834**	PrVF SwC–43 RNA1B	KX192389	5824 – 5842	reverse	sequencing	GTTCGAATCCTCCCTCTTG
**835**	PrVF SwC–43 RNA1B	KX192389	4343 – 4359	forward	sequencing	CCCGATTCAAAAGTTGC
**838**	PrVF SwC–43 RNA1B	KX192389	2556 – 2579	forward	sequencing	TTAGAAAGGGCATCTTGTATGTGT
**839**	PrVF SwC–43 RNA1A	KX192388	1307 – 1328	reverse	sequencing	TGTCTAAACACCCAATTCTGAG
**840**	PrVF SwC–43 RNA1A	KX192388	1307 – 1329	forward	sequencing	CTCAGAATTGGGTGTTTAGACAG
**841**	PrVF SwC–43 RNA1C	KX192390	267 – 285	forward	5' RACE	CACCTTCACTTTGGTTGCT
**850**	PrVF SwC–43 RNA2C	KX192393	326 – 343	reverse	5' RACE	ACGCTTACAGGGAGTGAG
**851**	PrVF SwC–43 RNA2C	KX192393	217 – 239	reverse	5' RACE	CAGCGTATGTTTCAAAGCCCGTA
**853**	PrVF SwC–43 RNA1B	KX192389	439 – 456	reverse	5' RACE	ATGTCCACCACCTCATCC
**854**	PrVF SwC–43 RNA1C	KX192390	269 – 291	reverse	5' RACE	ACCTCCAGCAACCAAAGTGAAGG
**857**	PrVF SwC–43 RNA1B	KX192389	1068 – 1088	forward	sequencing	TGGTCAAAGGATCATGCATGG
**870**	PrVF SwC–43 RNA2A	KX192391	1634 – 1655	reverse	sequencing	CTGCCATAGAGAAGGAAGCCAG
**871**	PrVF SwC–43 RNA1C	KX192390	23 – 48	forward	sequencing	CTTTCGATACCAGCTCTTCTTAAAGC
**927**	PrVF SwC–43 RNA1C	KX192390	3479 – 3501	forward	sequencing	TTTTTGATGTGGAGAGAGATCTT
**930**	CVF SwC–H RNA1B	MG925372	3556 – 3578	forward	sequencing	TCCTTTCATACAAGTATTATGCT
**931**	PrVF SwC–43 RNA1C	KX192390	3563 – 3586	forward	RNA1 universal	TTCCYGARATTGCYAAGATWGATG
**931**	PrVF SwC–43 RNA1A	KX192388	3575 – 3598	forward	RNA1 universal	TTCCYGARATTGCYAAGATWGATG
**931**	CVF SwC–H RNA1B	MG925372	3595 – 3618	forward	RNA1 universal	TTCCYGARATTGCYAAGATWGATG
**931**	PrVF SwC–43 RNA1C	KX192390	3563 – 3586	forward	RNA1 universal	TTCCYGARATTGCYAAGATWGATG
**931**	PrVF SwC–43 RNA1B	KX192389	3570 – 3593	forward	RNA1 universal	TTCCYGARATTGCYAAGATWGATG
**932**	PrVF SwC–43 RNA1A	KX192388	5059 – 5081	reverse	RNA1 universal	ATRGTKATRTGSADYTTKKCCAT
**932**	CVF SwC–H RNA1B	MG925372	5079 – 5101	reverse	RNA1 universal	ATRGTKATRTGSADYTTKKCCAT
**932**	PrVF SwC–43 RNA1C	KX192390	5047 – 5069	reverse	RNA1 universal	ATRGTKATRTGSADYTTKKCCAT
**932**	PrVF SwC–43 RNA1B	KX192389	5054 – 5076	reverse	RNA1 universal	ATRGTKATRTGSADYTTKKCCAT
**932**	PrVF SwC–43 RNA1C	KX192390	5047 – 5069	reverse	RNA1 universal	ATRGTKATRTGSADYTTKKCCAT
**933**	PrVF SwC–43 RNA1C	KX192390	5056 – 5078	reverse	sequencing	CCATCTGTAATGGTTATGTGCAA
**935**	CVF SwC–H RNA1B	MG925372	5145 – 5168	reverse	sequencing	GAGAAAATCGCACTTGTCAAGAGT
**950**	PrVF SwC–43 RNA2C	KX192393	3092 – 3114	forward	3' RACE, RNA2 universal	GGHHWVAYWCTYATGGCYAARTT
**950**	PrVF SwC–43 RNA2B	KX192392	3115 – 3137	forward	3' RACE, RNA2 universal	GGHHWVAYWCTYATGGCYAARTT
**950**	PrVF SwC–43 RNA2A	KX192391	3095 – 3117	forward	3' RACE, RNA2 universal	GGHHWVAYWCTYATGGCYAARTT
**951**	PrVF SwC–43 RNA1A	KX192388	5570 – 5591	forward	sequencing	CRAAYGACTATGTTGTTTGGCT
**951**	PrVF SwC–43 RNA1B	KX192389	5565 – 5586	forward	sequencing	CRAAYGACTATGTTGTTTGGCT
**952**	PrVF SwC–43 RNA1C	KX192390	4759 – 4778	reverse	RNA1 universal	GCCATBARNAGATTYTCTCG
**952**	PrVF SwC–43 RNA1B	KX192389	4766 – 4785	reverse	RNA1 universal	GCCATBARNAGATTYTCTCG
**952**	PrVF SwC–43 RNA1A	KX192388	4771 – 4790	reverse	RNA1 universal	GCCATBARNAGATTYTCTCG
**953**	PrVF SwC–43 RNA2C	KX192393	3269 – 3292	reverse	sequencing	GRAGARYCAAACAGAATARGTGTC
**953**	PrVF SwC–43 RNA2B	KX192392	3296 – 3319	reverse	sequencing	GRAGARYCAAACAGAATARGTGTC
**953**	CVF SwC–H RNA1B	MG925372	6020 – 6043	reverse	RNA2 universal	GRAGARYCAAACAGAATARGTGTC
**953**	PrVF SwC–43 RNA1A	KX192388	5994 – 6017	reverse	RNA2 universal	GRAGARYCAAACAGAATARGTGTC
**953**	PrVF SwC–43 RNA1B	KX192389	5996 – 6019	reverse	sequencing	GRAGARYCAAACAGAATARGTGTC
**953**	PrVF SwC–43 RNA2A	KX192391	3284 – 3307	reverse	RNA2 universal	GRAGARYCAAACAGAATARGTGTC
**954**	PrVF SwC–43 RNA2C	KX192393	1557 – 1577	forward	sequencing	CTGGTGAAATGACCCAGCGTG
**957**	PrVF SwC–43 RNA2C	KX192393	3234 – 3258	reverse	3' RACE	CTGGAAAATCTCCAGTAGTTCCAAT
**957**	PrVF SwC–43 RNA2C	KX192393	3234 – 3258	reverse	3' RACE	CTGGAAAATCTCCAGTAGTTCCAAT
**958**	PrVF SwC–43 RNA2C	KX192393	1308 – 1333	forward	sequencing	CAAATATTTCCATTCCGTTTGGTAGG

**959**	PrVF SwC–43 RNA2B	KX192392	1532 – 1557	forward	sequencing	AAGCTGTTTCTTCTTTGGAAGTTGAA
**960**	PrVF SwC–43 RNA2B	KX192392	3354 – 3376	reverse	3' RACE	CAGGTGGGACTCGAAATCCAACG
**960**	PrVF SwC–43 RNA1A	KX192388	6052 – 6074	reverse	3' RACE	CAGGTGGGACTCGAAATCCAACG
**960**	PrVF SwC–43 RNA1B	KX192389	6054 – 6076	reverse	3' RACE	CAGGTGGGACTCGAAATCCAACG
**961**	PrVF SwC–43 RNA2A	KX192391	1637 – 1664	forward	sequencing	GCTTCCTTCTCTATGGCAGATGTTATAT
**962**	PrVF SwC–43 RNA1B	KX192389	4548 – 4570	forward	sequencing	TTCTCATGAARARAAGRGATGTG
**962**	PrVF SwC–43 RNA1C	KX192390	4541 – 4563	forward	sequencing	TTCTCATGAARARAAGRGATGTG
**991**	PrVF SwC–43 RNA1C	KX192390	5869 – 5889	forward	sequencing	AGTTTGGGTTATCTTCCTGAA
**992**	PrVF SwC–43 RNA1A	KX192388	5321 – 5342	forward	sequencing	TAAACTTGCGAAGAAAAGCTCT
**993**	PrVF SwC–43 RNA1B	KX192389	5277 – 5297	forward	sequencing	TTTTGCGAGAACTTTTTCTAC
**994**	CVF SwC–H RNA1B	MG925372	5835 – 5853	forward	3' RACE	CGTTGTGGAGCCTTGGCTG
**123Up**	CVF SwC–G15 3 RNA1	LT991639	172-191	reverse	sequencing	ACTCTCACGTTGGTTGTAGG
**622Do**	CVF SwC–G15 3 RNA1	LT991639	656-679	forward	sequencing	CAAGAACATTGTAGACATGAGGAC
**422up**	CVF SwC–G15 3 RNA1	LT991639	482-502	reverse	sequencing	TTGCTTCTCAGTCTTCATCCT
**728do**	CVF SwC–G15 3 RNA1	LT991639	768-788	forward	sequencing	GCCAGGCCCATCACGACACAT
**622UP**	CVF SwC–G15 3 RNA1	LT991639	656-679	reverse	sequencing	GTCCTCATGTCTACAATGTTCTTG
**847do**	CVF SwC–G15 3 RNA1	LT991639	987-1006	forward	sequencing	CCAGTAGAGACATGCAGGAG
**826UP**	CVF SwC–G15 3 RNA1	LT991639	886-906	reverse	sequencing	ACATTTTCTATTGGGGCTGTT
**1118DO**	CVF SwC–G15 3 RNA1	LT991639	1158-1178	forward	sequencing	ATAAAAGCTGCCCATTGTTCT
**847Up**	CVF SwC–G15 3 RNA1	LT991639	987-1006	reverse	sequencing	CTCCTGCATGTCTCTACTGG
**1760Do**	CVF SwC–G15 3 RNA1	LT991639	1795-1816	forward	sequencing	TTTGTGTGAGTGAGCTTTTTCC
**1568UP**	CVF SwC–G15 3 RNA1	LT991639	1628-1649	reverse	sequencing	GCAGTGTTTTCATAGGAAGTGA
**1850DO**	CVF SwC–G15 3 RNA1	LT991639	1628-1649	forward	sequencing	CGACGATAGCCAGACCAAAA
**1760UP**	CVF SwC–G15 3 RNA1	LT991639	1795-1816	reverse	sequencing	GGAAAAAGCTCACTCACACAAA
**2546DO**	CVF SwC–G15 3 RNA1	LT991639	2602-2626	forward	sequencing	CCATTTGATGATTAGGCAGAACA
**2464UP**	CVF SwC–G15 3 RNA1	LT991639	2524-2544	reverse	sequencing	GTGACTCTTGCTGGGATTGTC
**2671DO**	CVF SwC–G15 3 RNA1	LT991639	2713-2733	forward	sequencing	CTTTCGGGTGTAGTCATCAGG
**2546Up**	CVF SwC–G15 3 RNA1	LT991639	2602-2626	reverse	sequencing	TGTTCTGCCTAATCATCAAATGG
**3299Do**	CVF SwC–G15 3 RNA1	LT991639	3338-3357	forward	sequencing	CCGATAGACAGCCAACTCAC
**3140Up**	CVF SwC–G15 3 RNA1	LT991639	3198-3220	reverse	sequencing	TGTTCTGCCTAATCATCAAATGG
**3865Do**	CVF SwC–G15 3 RNA1	LT991639	3899-3923	forward	sequencing	GTTATTACGCTGGGTATCTTTTCAC
**3530Up**	CVF SwC–G15 3 RNA1	LT991639	3588-3609	reverse	sequencing	TGCTGAAACTATAGACGGTGAC
**4391Do**	CVF SwC–G15 3 RNA1	LT991639	4429-4449	forward	sequencing	GAATTTCATGCGCACAACAAG
**4263Up**	CVF SwC–G15 3 RNA1	LT991639	4321-4341	reverse	sequencing	CCTGAACTGGTTGGAATCGAG
**5103Do**	CVF SwC–G15 3 RNA1	LT991639	5140-5160	forward	sequencing	TTCAAGGTTACGCGTGTTGAC
**4939UP**	CVF SwC–G15 3 RNA1	LT991639	4979-5001	reverse	sequencing	AAATGCACATCACCATCACTGAT
**CVF-3UTR do**	CVF SwC–G15 3 RNA1	LT991639	5925-5945	forward	sequencing	AGCAGGCYGAAGAGCCAAAC
**141UP**	CVF SwC–G15 3 RNA2	LT991640	141-161	reverse	sequencing	GTGGTTACCAAAGCCCTCATT
**712DO**	CVF SwC–G15 3 RNA2	LT991640	800-820	forward	sequencing	ACATTTTCAATTCGCCTGGTG
**571Up**	CVF SwC–G15 3 RNA2	LT991640	679-699	reverse	sequencing	CCTACCGTGAAACAGTTCCTC
**1325Do**	CVF SwC–G15 3 RNA2	LT991640	1411-1433	forward	sequencing	AACAGATCTTGAGAAGGGTATGG
**951Up**	CVF SwC–G15 3 RNA2	LT991640	1059-1080	reverse	sequencing	CAAGATGCGCGTAATGCTTTTC
**1850Do**	CVF SwC–G15 3 RNA2	LT991640	1941-1961	forward	sequencing	ACAAGCGGGATTCCAAAAGAC
**1724Up**	CVF SwC–G15 3 RNA2	LT991640	1835-1854	reverse	sequencing	TTTGATTCCTCCTGCGTGTG
**2515Do**	CVF SwC–G15 3 RNA2	LT991640	2605-2626	forward	sequencing	ACACTAGAAGTCACGCTATCAC
**2425UP**	CVF SwC–G15 3 RNA2	LT991640	2539-2561	reverse	sequencing	CCAATTCTGCTAGTGTCAATCAT
**R2-3END R**	CVF SwC–G15 3 RNA2	LT991640	3887-3906	forward	sequencing	TGGGCTTTCACCATTCTCA

Supplementary Table 1 Expected sizes of PCR amplified bands with the above-listed primers

Target sequence	Accession number	Fwd. primer name	Rev. primer name	Fragment length, bp
**CVF SwC–G15 3 RNA2**	LT991640	571Up	1325Do	755
**CVF SwC–G15 3 RNA2**	LT991640	571Up	1850Do	1277
**CVF SwC–G15 3 RNA2**	LT991640	571Up	2515Do	1942
**CVF SwC–G15 3 RNA2**	LT991640	951Up	1850Do	897
**CVF SwC–G15 3 RNA2**	LT991640	951Up	2515Do	1562
**CVF SwC–G15 3 RNA2**	LT991640	1724Up	2515Do	792
**CVF SwC–G15 3 RNA1**	LT991639	123Up	847do	835
**CVF SwC–G15 3 RNA1**	LT991639	123Up	1118DO	1007
**CVF SwC–G15 3 RNA1**	LT991639	123Up	1760Do	1645
**CVF SwC–G15 3 RNA1**	LT991639	123Up	1850DO	1739

Target sequence	Accession number	Fwd. primer name	Rev. primer name	Fragment length, bp
**CVF SwC–G15 3 RNA1**	LT991639	123Up	2671DO	2562
**CVF SwC–G15 3 RNA1**	LT991639	422up	1760Do	1335
**CVF SwC–G15 3 RNA1**	LT991639	422up	1850DO	1429
**CVF SwC–G15 3 RNA1**	LT991639	422up	2671DO	2252
**CVF SwC–G15 3 RNA1**	LT991639	422up	2546DO	2739
**CVF SwC–G15 3 RNA1**	LT991639	422up	3299Do	2876
**CVF SwC–G15 3 RNA1**	LT991639	622UP	1760Do	1161
**CVF SwC–G15 3 RNA1**	LT991639	622UP	1850DO	1255
**CVF SwC–G15 3 RNA1**	LT991639	622UP	2671DO	2078
**CVF SwC–G15 3 RNA1**	LT991639	622UP	2546DO	2565
**CVF SwC–G15 3 RNA1**	LT991639	622UP	3299Do	2702
**CVF SwC–G15 3 RNA1**	LT991639	826UP	1760Do	931
**CVF SwC–G15 3 RNA1**	LT991639	826UP	1850DO	1025
**CVF SwC–G15 3 RNA1**	LT991639	826UP	2671DO	1848
**CVF SwC–G15 3 RNA1**	LT991639	826UP	2546DO	2335
**CVF SwC–G15 3 RNA1**	LT991639	826UP	3299Do	2472
**CVF SwC–G15 3 RNA1**	LT991639	1568UP	2671DO	1106
**CVF SwC–G15 3 RNA1**	LT991639	1568UP	2546DO	1593
**CVF SwC–G15 3 RNA1**	LT991639	1568UP	3299Do	1730
**CVF SwC–G15 3 RNA1**	LT991639	1568UP	3865Do	2296
**CVF SwC–G15 3 RNA1**	LT991639	1568UP	4391Do	2822
**CVF SwC–G15 3 RNA1**	LT991639	2464UP	3299Do	834
**CVF SwC–G15 3 RNA1**	LT991639	2464UP	3865Do	1400
**CVF SwC–G15 3 RNA1**	LT991639	2464UP	4391Do	1926
**CVF SwC–G15 3 RNA1**	LT991639	2464UP	5103Do	2637
**CVF SwC–G15 3 RNA1**	LT991639	2546Up	3865Do	726
**CVF SwC–G15 3 RNA1**	LT991639	2546Up	4391Do	1252
**CVF SwC–G15 3 RNA1**	LT991639	2546Up	5103Do	1963
**CVF SwC–G15 3 RNA1**	LT991639	3140Up	3865Do	726
**CVF SwC–G15 3 RNA1**	LT991639	3140Up	4391Do	1252
**CVF SwC–G15 3 RNA1**	LT991639	3140Up	5103Do	1963
**CVF SwC–G15 3 RNA1**	LT991639	3530Up	4391Do	862
**CVF SwC–G15 3 RNA1**	LT991639	3530Up	5103Do	1573
**CVF SwC–G15 3 RNA1**	LT991639	4263Up	5103Do	840
**CVF SwC–H RNA2**	MG925373	1037	1409	863
**CVF SwC–H RNA2**	MG925373	1037	1038	3843
**CVF SwC–H RNA2**	MG925373	1039	1038	3709
**CVF SwC–H RNA2**	MG925373	1562	1038	2234
**CVF SwC–H RNA1B**	MG925372	1037	1038	6174
**CVF SwC–H RNA1B**	MG925372	930	952	1255
**CVF SwC–H RNA1B**	MG925372	930	932	1546
**CVF SwC–H RNA1B**	MG925372	930	935	1613
**CVF SwC–H RNA1B**	MG925372	930	953	2488
**CVF SwC–H RNA1B**	MG925372	930	1038	2621
**CVF SwC–H RNA1B**	MG925372	931	952	1216
**CVF SwC–H RNA1B**	MG925372	931	932	1507
**CVF SwC–H RNA1B**	MG925372	931	935	1574
**CVF SwC–H RNA1B**	MG925372	931	953	2449
**CVF SwC–H RNA1B**	MG925372	931	1038	2582
**PrVF SwC–43 RNA1A**	KX192388	1037	1019	821
**PrVF SwC–43 RNA1A**	KX192388	1037	1018	877
**PrVF SwC–43 RNA1A**	KX192388	1037	1034	1013
**PrVF SwC–43 RNA1A**	KX192388	1037	839	1324
**PrVF SwC–43 RNA1A**	KX192388	1037	1038	6147
**PrVF SwC–43 RNA1A**	KX192388	1083	1019	723
**PrVF SwC–43 RNA1A**	KX192388	1083	1018	779
**PrVF SwC–43 RNA1A**	KX192388	1083	1034	915
**PrVF SwC–43 RNA1A**	KX192388	1083	839	1226
**PrVF SwC–43 RNA1A**	KX192388	1083	1038	6049
**PrVF SwC–43 RNA1A**	KX192388	840	821	2956
**PrVF SwC–43 RNA1A**	KX192388	931	820	857

Target sequence	Accession number	Fwd. primer name	Rev. primer name	Fragment length, bp
**PrVF SwC–43 RNA1A**	KX192388	931	952	1216
**PrVF SwC–43 RNA1A**	KX192388	931	932	1507
**PrVF SwC–43 RNA1A**	KX192388	931	953	2443
**PrVF SwC–43 RNA1A**	KX192388	931	960	2500
**PrVF SwC–43 RNA1A**	KX192388	931	1038	2577
**PrVF SwC–43 RNA1A**	KX192388	823	820	781
**PrVF SwC–43 RNA1A**	KX192388	823	952	1140
**PrVF SwC–43 RNA1A**	KX192388	823	932	1431
**PrVF SwC–43 RNA1A**	KX192388	823	953	2367
**PrVF SwC–43 RNA1A**	KX192388	823	960	2424
**PrVF SwC–43 RNA1A**	KX192388	823	1038	2501
**PrVF SwC–43 RNA1A**	KX192388	822	952	808
**PrVF SwC–43 RNA1A**	KX192388	822	932	1099
**PrVF SwC–43 RNA1A**	KX192388	822	953	2035
**PrVF SwC–43 RNA1A**	KX192388	822	960	2092
**PrVF SwC–43 RNA1A**	KX192388	822	1038	2169
**PrVF SwC–43 RNA1A**	KX192388	992	960	754
**PrVF SwC–43 RNA1A**	KX192388	992	1038	831
**PrVF SwC–43 RNA2A**	KX192391	1037	1010	822
**PrVF SwC–43 RNA2A**	KX192391	1037	1014	906
**PrVF SwC–43 RNA2A**	KX192391	1037	830	1418
**PrVF SwC–43 RNA2A**	KX192391	1037	870	1651
**PrVF SwC–43 RNA2A**	KX192391	954	953	1748
**PrVF SwC–43 RNA2A**	KX192391	1037	1038	3586
**PrVF SwC–43 RNA2A**	KX192391	954	1038	2031
**PrVF SwC–43 RNA2A**	KX192391	961	953	1671
**PrVF SwC–43 RNA2A**	KX192391	961	1038	1954
**PrVF SwC–43 RNA1B**	KX192389	1037	1017	814
**PrVF SwC–43 RNA1B**	KX192389	1037	1018	872
**PrVF SwC–43 RNA1B**	KX192389	1037	1034	1008
**PrVF SwC–43 RNA1B**	KX192389	1083	1017	721
**PrVF SwC–43 RNA1B**	KX192389	1083	1018	779
**PrVF SwC–43 RNA1B**	KX192389	1083	1038	6056
**PrVF SwC–43 RNA1B**	KX192389	841	1034	739
**PrVF SwC–43 RNA1B**	KX192389	838	952	2230
**PrVF SwC–43 RNA1B**	KX192389	838	932	2521
**PrVF SwC–43 RNA1B**	KX192389	931	952	1216
**PrVF SwC–43 RNA1B**	KX192389	931	932	1507
**PrVF SwC–43 RNA1B**	KX192389	931	834	2273
**PrVF SwC–43 RNA1B**	KX192389	931	953	2450
**PrVF SwC–43 RNA1B**	KX192389	931	960	2507
**PrVF SwC–43 RNA1B**	KX192389	931	1038	2584
**PrVF SwC–43 RNA1B**	KX192389	962	834	1295
**PrVF SwC–43 RNA1B**	KX192389	962	953	1472
**PrVF SwC–43 RNA1B**	KX192389	962	960	1529
**PrVF SwC–43 RNA1B**	KX192389	962	1038	1606
**PrVF SwC–43 RNA1B**	KX192389	993	953	743
**PrVF SwC–43 RNA1B**	KX192389	993	960	800
**PrVF SwC–43 RNA1B**	KX192389	993	1038	877
**PrVF SwC–43 RNA2B**	KX192392	1037	1038	3449
**PrVF SwC–43 RNA2B**	KX192392	1037	1010	842
**PrVF SwC–43 RNA2B**	KX192392	1037	830	1438
**PrVF SwC–43 RNA2B**	KX192392	829	818	1748
**PrVF SwC–43 RNA2B**	KX192392	829	953	1894
**PrVF SwC–43 RNA2B**	KX192392	829	960	1951
**PrVF SwC–43 RNA2B**	KX192392	829	1038	2028
**PrVF SwC–43 RNA2B**	KX192392	959	818	1642
**PrVF SwC–43 RNA2B**	KX192392	959	953	1788
**PrVF SwC–43 RNA2B**	KX192392	959	960	1845
**PrVF SwC–43 RNA2B**	KX192392	959	1038	1922
**PrVF SwC–43 RNA2B**	KX192392	954	818	1594

Target sequence	Accession number	Fwd. primer name	Rev. primer name	Fragment length, bp
**PrVF SwC–43 RNA2B**	KX192392	954	953	1740
**PrVF SwC–43 RNA2B**	KX192392	954	960	1797
**PrVF SwC–43 RNA2B**	KX192392	954	1038	1874
**PrVF SwC–43 RNA2B**	KX192392	819	960	756
**PrVF SwC–43 RNA2B**	KX192392	819	1038	833
**PrVF SwC–43 RNA1C**	KX192390	1037	1018	865
**PrVF SwC–43 RNA1C**	KX192390	1037	1034	1001
**PrVF SwC–43 RNA1C**	KX192390	871	1018	847
**PrVF SwC–43 RNA1C**	KX192390	871	1034	983
**PrVF SwC–43 RNA1C**	KX192390	1083	1018	775
**PrVF SwC–43 RNA1C**	KX192390	1083	1034	911
**PrVF SwC–43 RNA1C**	KX192390	841	1034	739
**PrVF SwC–43 RNA1C**	KX192390	1150	952	1371
**PrVF SwC–43 RNA1C**	KX192390	1150	932	1662
**PrVF SwC–43 RNA1C**	KX192390	1150	933	1671
**PrVF SwC–43 RNA1C**	KX192390	927	952	1300
**PrVF SwC–43 RNA1C**	KX192390	927	932	1591
**PrVF SwC–43 RNA1C**	KX192390	927	933	1600
**PrVF SwC–43 RNA1C**	KX192390	931	952	1216
**PrVF SwC–43 RNA1C**	KX192390	931	932	1507
**PrVF SwC–43 RNA1C**	KX192390	931	933	1516
**PrVF SwC–43 RNA2C**	KX192393	1037	1010	819
**PrVF SwC–43 RNA2C**	KX192393	1037	1038	3740
**PrVF SwC–43 RNA2C**	KX192393	1037	830	1415
**PrVF SwC–43 RNA2C**	KX192393	827	1009	703
**PrVF SwC–43 RNA2C**	KX192393	827	830	1200
**PrVF SwC–43 RNA2C**	KX192393	958	957	1951
**PrVF SwC–43 RNA2C**	KX192393	958	953	1985
**PrVF SwC–43 RNA2C**	KX192393	958	1038	2437
**PrVF SwC–43 RNA2C**	KX192393	829	957	1856
**PrVF SwC–43 RNA2C**	KX192393	829	953	1890
**PrVF SwC–43 RNA2C**	KX192393	829	1038	2342
**PrVF SwC–43 RNA2C**	KX192393	954	957	1702
**PrVF SwC–43 RNA2C**	KX192393	954	953	1736
**PrVF SwC–43 RNA2C**	KX192393	954	1038	2188
